# Trends in Glucagon-Like Peptide-1 Agonist Use and BMI Among Obese Adults With Type 2 Diabetes: Analysis of the 2010-2015 National Ambulatory Medical Care Survey (NAMCS), Adjusted for Cardiovascular Comorbidity

**DOI:** 10.7759/cureus.90054

**Published:** 2025-08-14

**Authors:** Victoria Eneh, Naomi F Chatelain, Okelue E Okobi, Efeturi M Okorigba, Ugochukwu P Chukwuebuni, Tochukwu W Okahia, Rita A Bampoh, Edamisan J Fanegan, Chioma C Ubajaka, Adebola Y Afolayan, Sunday O Arifayan, Kenechukwu Ijeomah

**Affiliations:** 1 Medicine, Ebonyi State University, Abakaliki, NGA; 2 Medicine, Avalon University School of Medicine, Willemstad, CUW; 3 Family Medicine, Larkin Community Hospital Palm Springs Campus, Miami, USA; 4 Family Medicine, IMG Research Academy and Consulting LLC, Homestead, USA; 5 Internal Medicine, West Virginia University, Morgantown, USA; 6 Internal Medicine, Port Antonio Hospital, Port Antonio, JAM; 7 Psychiatry, Leeds and York Partnership NHS Foundation Trust, Leeds, GBR; 8 Internal Medicine, University of Ghana Medical Centre, Accra, GHA; 9 Internal Medicine, RWJBarnabas Health, Newark, USA; 10 Internal Medicine, Igbinedion University Okada, Benin City, NGA; 11 Internal Medicine, Triboro Center for Nursing and Rehabilitation, New York, USA; 12 Internal Medicine, University of Ilorin College of Health Sciences, Ilorin, NGA; 13 Family Medicine, University of Ilorin Teaching Hospital, Ilorin, NGA; 14 Anesthesiology and Critical Care, Marigold Hospital and Critical Care Centre, Lagos, NGA

**Keywords:** bmi, glp-1 receptor agonists, namcs, obesity, prescription trends, type 2 diabetes

## Abstract

Background and objective

Glucagon-like peptide-1 (GLP-1) receptor agonists are increasingly used to manage type 2 diabetes (T2D) and promote weight loss; however, their real-world use remains undercharacterized. The objective of this study was to assess trends in GLP-1 agonist prescriptions and their association with BMI among obese adults with T2D in US outpatient settings.

Methods

We analyzed data from the National Ambulatory Medical Care Survey for the years 2010-2015. Adults diagnosed with both obesity and T2D were included. GLP-1 use was identified using Multum drug codes. Weighted descriptive statistics, design-based F-tests, t-tests, and linear regression were used to assess associations between GLP-1 use and BMI, adjusting for demographics, insurance, cardiovascular disease, and year.

Results

Of 121,266 adult outpatient visits analyzed, only 0.13% involved a GLP-1 prescription, reflecting limited use during the study period. GLP-1 users had a significantly higher BMI compared with nonusers (β = 5.98, p < 0.001). Use of GLP-1s showed no consistent increase over time. Several factors, including age, sex, race/ethnicity, insurance type, and cardiovascular comorbidity, were significantly associated with BMI.

Conclusions

GLP-1 agonists were underutilized between 2010 and 2015 and were more likely to be prescribed to patients with higher BMI. These findings underscore the need to expand access to and guideline-based prescribing of these agents to optimize diabetes and obesity care.

## Introduction

Type 2 diabetes mellitus (T2DM) and obesity are closely linked public health challenges in the US, both contributing substantially to the nation’s chronic disease burden [1]. According to the CDC, 42% of US adults are obese, and an estimated 11% live with diagnosed diabetes, mostly type 2 [2]. Obesity increases the likelihood of T2DM and makes its treatment more challenging by exacerbating glycemic control efforts and predisposing patients to complications such as hypertension, dyslipidemia, and cardiovascular disease (CVD) [3]. These comorbidities have a cumulative effect on morbidity and mortality, requiring novel, multifaceted treatment strategies [4]. During this health crisis, glucagon-like peptide-1 (GLP-1) receptor agonists have emerged as an effective pharmacological approach [5].

GLP-1 receptor agonists (GLP-1RAs), initially developed for glycemic control in T2DM, have demonstrated additional benefits, including weight loss, appetite suppression, and cardioprotection [6,7]. These drugs mimic the effects of endogenous GLP-1 by enhancing insulin release, inhibiting glucagon secretion, and slowing gastric emptying [8]. The advent of GLP-1 agonists has addressed the dual need for glycemic control and weight reduction in obese patients with diabetes [9,10]. In the early 2010s, agents such as exenatide, liraglutide, and dulaglutide became increasingly common in diabetes management regimens [11]. Clinical trials and real-world evidence show that, in addition to lowering HbA1c levels, GLP-1 agonists significantly reduce BMI, one of the primary therapeutic targets in obese patients with T2DM [12,13].

Despite their potential, uptake of GLP-1 agonists in clinical practice has been inconsistent [14]. Prescription patterns may be influenced by several factors, including patient demographics (e.g., age, sex, and race/ethnicity), socioeconomic factors (e.g., insurance coverage), physician prescribing habits, and comorbid conditions such as CVD [15,16]. Notably, the US FDA has approved some GLP-1RAs for reducing cardiovascular risk in patients with established atherosclerotic CVD, further supporting their clinical utility and increasing their use [17,18]. Nevertheless, disparities in prescribing may exist among different subgroups of the population.

Understanding these trends is particularly important when examined using large-scale, nationally representative datasets. The National Ambulatory Medical Care Survey (NAMCS) provides valuable insights into real-world prescribing patterns of GLP-1 agonists and their associations with obesity and cardiovascular risk factors in US adults with T2DM [19]. Between 2010 and 2015, the period under review, there was growing attention to weight-focused diabetes treatment and increasing awareness of the cardiometabolic benefits of GLP-1 agonists [20]. However, gaps remain in understanding how these developments have translated into clinical practice, particularly regarding demographic and clinical variations.

This study examines the association between GLP-1 agonist use and BMI among obese adults with T2DM while adjusting for cardiovascular comorbidities and other key patient characteristics, including age, sex, race/ethnicity, and insurance type. It also evaluates national prescribing trends of GLP-1 agonists over time.

## Materials and methods

Study design and data source

This study employed a retrospective, cross-sectional design using data from the NAMCS covering the years 2010 through 2015 [20]. NAMCS uses a multistage probability sampling design to collect data from visits to nonfederal, office-based physicians primarily engaged in direct patient care. The dataset includes information on patient demographics, diagnoses, prescribed medications, vital signs, services provided, and physician characteristics. A cross-sectional approach was selected to evaluate prescribing practices and associated patient characteristics at the national level across multiple time points. This design is well suited for detecting patterns in medication use over time and for assessing how clinical outcomes, such as BMI, may vary by treatment type in routine care settings.

Study population

The study sample was restricted to adult patients aged 18 years or older with a recorded diagnosis of T2DM and documented obesity. T2DM diagnosis was identified using International Classification of Diseases, Ninth Revision, Clinical Modification (ICD-9-CM) codes 250.xx. Obesity status was based on a predefined NAMCS variable coded as “Yes” or “No.” The final analytic sample included adult visits involving obese patients with T2DM.

Only visits with both a recorded medication list and a BMI measurement at the time of the encounter were eligible for analysis. Each NAMCS visit represents a single ambulatory care encounter and is weighted to produce national population estimates. No missing data handling procedures were required, as all retained records were complete for the variables of interest after applying the inclusion criteria.

Study variables

The primary outcome was BMI, measured in kilograms per square meter (kg/m²) and recorded during the patient visit. The main exposure was the prescription of a GLP-1RA, identified using Multum drug codes listed in the DRUGID1 to DRUGID8 variables. GLP-1 use was coded as a binary variable (yes = 1, no = 0). To assess prescribing trends, a survey year variable was created and used in temporal analyses.

Demographic covariates included age (continuous), sex (male/female), and race/ethnicity. Socioeconomic status was approximated using the expected source of payment (private, Medicaid, and self-pay/other). Clinical covariates included the presence of CVD. CVD was defined as the presence of ischemic heart disease or congestive heart failure, based on diagnosis fields in NAMCS. A composite binary variable captured the presence of any CVD condition (yes = 1, no = 0), allowing adjustment for cardiovascular risk, a potential confounder affecting both GLP-1 prescribing patterns and BMI.

Statistical analysis

All analyses were conducted using Stata version 18 (StataCorp LLC, College Station, TX, US), incorporating survey weights, strata, and primary sampling units to account for NAMCS’s complex survey design and to ensure nationally representative estimates. Descriptive statistics summarized patient and visit characteristics, stratified by GLP-1 agonist prescription status. Continuous variables were reported as weighted means with standard deviations, while categorical variables were expressed as weighted percentages.

National trends in GLP-1 prescribing from 2010 to 2015 were assessed by calculating the proportion of visits involving a GLP-1 prescription for each survey year. The association between GLP-1 agonist use and BMI was evaluated using a multivariable survey-weighted linear regression model, with BMI as the dependent variable and GLP-1 use as the primary independent variable. The model was adjusted for age, sex, race/ethnicity, insurance type, and CVD. Multicollinearity was assessed using variance inflation factors (VIFs) from a standard linear regression model including all covariates; all VIF values were below 3.1 (mean VIF = 1.80), indicating no evidence of problematic multicollinearity.

Ethical considerations

This study was based entirely on secondary analysis of publicly available, de-identified data from NAMCS. As such, it is exempt from institutional review board oversight under the US Department of Health and Human Services policy for research using existing public datasets (45 CFR 46.104(d)(4)). No individual consent or ethical approval was required.

## Results

Table 1 presents the distribution of demographic and clinical characteristics among obese adults with type 2 diabetes, stratified by GLP-1 agonist (exenatide) prescription status, based on weighted estimates from the NAMCS for 2010-2015. Design-based F-tests and t-tests were used to compare characteristics between GLP-1 users and nonusers, accounting for the complex survey design.

**Table 1:** Survey-weighted patient characteristics by GLP-1 agonist use among obese adults with type 2 diabetes, NAMCS 2010-2015 (N = 2,651,676,034 visits) Values are weighted national estimates from the NAMCS, 2010-2015. Statistical comparisons are based on survey-weighted t-tests (for continuous variables) and design-based F-tests (for categorical variables). -: intentionally left blank CVD, cardiovascular disease; GLP-1, glucagon-like peptide-1; NAMCS, National Ambulatory Medical Care Survey

Characteristic	GLP-1 users (N = 3,880,935)	Nonusers (N = 2,647,795,098)	F-test/T-test	p-Value
BMI (kg/m²), mean ± SD	35.15 ± 7.14	29.11 ± 6.88	t = -5.80	<0.001
Patient age (years), mean ± SD	59.85 ± 10.15	55.55 ± 17.79	t = -4.23	<0.001
Sex (%)	-	-	F = 0.91	0.34
Male	1,804,360 (0.17%)	1,081,702,756 (99.83%)	-	-
Female	2,076,575 (0.13%)	1,566,092,341 (99.87%)	-	-
Insurance type (%)	-	-	F = 0.43	0.64
Medicaid	257,913 (0.10%)	252,552,962 (99.90%)	-	-
Private	2,354,078 (0.15%)	1,608,566,464 (99.85%)	-	-
Self-pay/other	1,268,943 (0.16%)	786,675,671 (99.84%)	-	-
CVD (%)	-	-	F = 1.01	0.32
No	3,546,684 (0.14%)	2,501,488,717 (99.86%)	-	-
Yes	334,250 (0.23%)	146,306,381 (99.77%)	-	-
Race/ethnicity (%)	-	-	F = 0.93	0.42
Non-Hispanic White	3,000,454 (0.16%)	1,903,807,112 (99.84%)	-	-
Non-Hispanic Black	518,282 (0.17%)	301,950,153 (99.83%)	-	-
Hispanic	213,071 (0.07%)	296,332,980 (99.93%)	-	-
Non-Hispanic other	149,126 (0.10%)	145,704,851 (99.90%)	-	-

The mean BMI was significantly higher among GLP-1 agonist users (35.15 ± 7.14 kg/m²) compared with nonusers (29.11 ± 6.88 kg/m²), with a t-test indicating a statistically significant difference (t = -5.80, p < 0.001). Similarly, GLP-1 users were older on average (59.85 ± 10.15 years) than nonusers (55.55 ± 17.79 years), and this difference was also statistically significant (t = -4.23, p < 0.001).

In terms of sex, 1,804,360 (0.17%) of GLP-1 users were male and 2,076,575 (0.13%) were female. Among nonusers, 1,081,702,756 (99.83%) were male and 1,566,092,341 (99.87%) were female. However, the difference in sex distribution between users and nonusers was not statistically significant (F = 0.91, p = 0.34).

Insurance type also did not differ significantly by GLP-1 use (F = 0.43, p = 0.64). Among users, the most common insurance type was private insurance (2,354,078; 0.15%), followed by self-pay/other (1,268,943; 0.16%) and Medicaid (257,913; 0.10%).

Regarding CVD, 334,250 (0.23%) of GLP-1 users had a CVD diagnosis compared with 146,306,381 (99.77%) of nonusers. Among those without CVD, 3,546,684 (0.14%) were GLP-1 users and 2,501,488,717 (99.86%) were nonusers. These differences were not statistically significant (F = 1.01, p = 0.32).

Race and ethnicity distributions also did not differ significantly by GLP-1 use (F = 0.93, p = 0.42). Among GLP-1 users, the majority were non-Hispanic White (3,000,454; 0.16%), followed by non-Hispanic Black (518,282; 0.17%), Hispanic (213,071; 0.07%), and non-Hispanic other (149,126; 0.10%).

**Figure 1:**
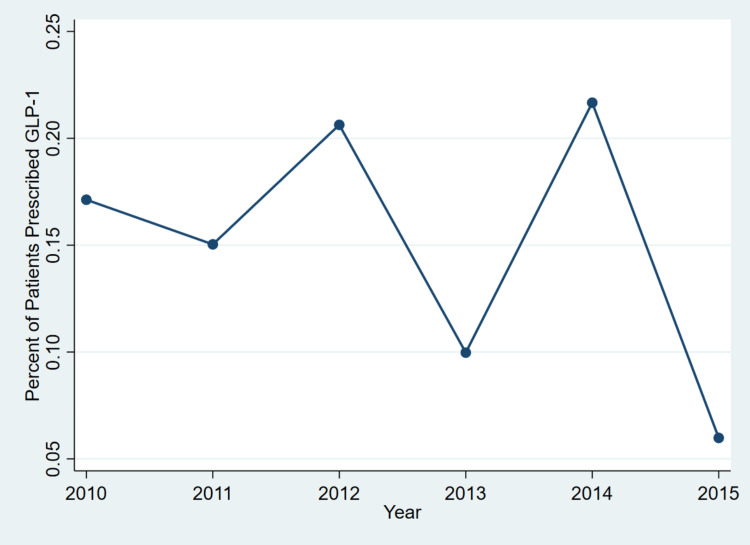
Trends in GLP-1 agonist (exenatide) prescriptions among obese adults with type 2 diabetes in US outpatient visits, 2010-2015 This figure shows the annual percentage of outpatient visits involving a GLP-1RA (exenatide) prescription among obese adults with type 2 diabetes in the US, based on NAMCS data from 2010 to 2015. Although the prescription rate varied across the years, no consistent upward trend was observed. GLP-1, glucagon-like peptide-1; GLP-1RA, glucagon-like peptide-1 receptor agonist; NAMCS, National Ambulatory Medical Care Survey

Figure 1 presents the annual percentage of obese adults with type 2 diabetes who were prescribed GLP-1RAs (exenatide) between 2010 and 2015, based on nationally representative NAMCS data. This trend analysis illustrates changes in the real-world clinical uptake of GLP-1 therapy over time.

Overall, the figure shows that the prescription of GLP-1RAs among obese adults with type 2 diabetes remained low throughout 2010-2015, with no consistent upward trend. The highest prescription rate occurred in 2014 (0.22%), followed closely by 2012 (0.21%) and 2010 (0.17%). The lowest rate was in 2015 (0.06%), suggesting a potential decline in exenatide use or a shift toward newer therapies not captured in this dataset.

Despite clinical endorsement and availability during this period, the adoption of GLP-1 agonists in outpatient practice appears limited. These persistently low prescribing rates may reflect barriers such as high medication costs, restrictive insurance coverage, clinical inertia, or lack of provider familiarity and comfort with newer injectable therapies.

Table 2 presents the results of a multivariable linear regression model assessing the association between GLP-1 agonist use (exenatide) and BMI while adjusting for demographic, clinical, and temporal covariates. The analysis includes data from 121,266 patient visits, representing a nationally weighted sample of obese adults with type 2 diabetes in the US between 2010 and 2015.

**Table 2:** Survey-weighted multivariable linear regression of BMI on GLP-1 use and covariates among obese adults with type 2 diabetes, NAMCS 2010-2015 Reference categories: male (sex), Medicaid (insurance), non-Hispanic White (race/ethnicity), and year 2010 (survey year) -: intentionally left blank CVD, cardiovascular disease; GLP-1, glucagon-like peptide-1; NAMCS, National Ambulatory Medical Care Survey

Variable	Coefficient (β)	SE
GLP-1 users	5.979	1.053
Patient age (years)	-0.009	0.003
Sex		
Female	-0.363	0.076
Any CVD	0.700	0.182
Insurance type		
Private	-0.747	0.139
Self-pay/other	-0.771	0.181
Race/ethnicity		
Non-Hispanic Black	1.848	0.138
Hispanic	0.211	0.153
Non-Hispanic other	-2.992	0.332
Survey year		
2011	0.673	0.183
2012	0.669	0.148
2013	0.657	0.155
2014	0.852	0.162
2015	0.646	0.219
Constant	29.774	0.249
Observations	121,266	-
R²	0.024	-

GLP-1 agonist use was associated with a statistically significant increase in BMI of 5.98 units (β = 5.979, SE = 1.053, p < 0.001). Although GLP-1 agonists are generally prescribed to promote weight loss, this finding may reflect clinical prescribing patterns in which providers are more likely to initiate these medications in patients with more severe obesity, leading to a higher average BMI among users.

Age was negatively associated with BMI; each additional year of age corresponded to a 0.009-unit decrease in BMI (β = -0.009, SE = 0.003, p < 0.001). Female patients had significantly lower BMI than male patients (β = -0.363, SE = 0.076, p < 0.001).

Patients with CVD had significantly higher BMI than those without CVD (β = 0.700, SE = 0.182, p < 0.001). Compared with patients insured through Medicaid (reference group), those with private insurance (β = -0.747, SE = 0.139, p < 0.001) or self-pay/other coverage (β = -0.771, SE = 0.181, p < 0.001) had significantly lower BMI.

Race and ethnicity were also significant predictors. Non-Hispanic Black patients had significantly higher BMI (β = 1.848, SE = 0.138, p < 0.001), whereas non-Hispanic other patients had significantly lower BMI (β = -2.992, SE = 0.332, p < 0.001) compared with non-Hispanic Whites. The difference for Hispanic patients was not statistically significant (β = 0.211, SE = 0.153, p = 0.17).

In terms of time trends, BMI was significantly higher in all survey years compared with the baseline year (2010), with the largest increase observed in 2014 (β = 0.852, SE = 0.162, p < 0.001) and the smallest in 2015 (β = 0.646, SE = 0.219, p < 0.01).

## Discussion

This nationally representative analysis of patient visits among obese adults with type 2 diabetes in the US from 2010 to 2015 provides important insights into the real-world use of GLP-1RAs, particularly exenatide, and their association with BMI. Despite clinical evidence from Castellana et al. [12] and Hu et al. [13] demonstrating the efficacy of GLP-1 agonists in promoting weight loss and glycemic control, our findings reveal persistently low prescription rates during the study period. Moreover, GLP-1 use was associated with significantly higher BMI values, a counterintuitive result that may reflect provider prescribing behaviors rather than treatment effect.

In our adjusted regression model, GLP-1 users had a BMI 5.98 units higher than nonusers, even after accounting for age, sex, CVD status, insurance type, race/ethnicity, and survey year. While GLP-1 agonists are clinically indicated for weight reduction, this finding likely reflects selection bias, wherein providers may preferentially prescribe GLP-1s to patients with more severe obesity or to those who have not responded to other treatment options. Additionally, because BMI was assessed cross-sectionally, the timing of prescription relative to weight change could not be determined, limiting causal inference. This highlights the complexity of real-world prescribing, where GLP-1RAs, despite their known metabolic and emerging neuroprotective benefits, remain underutilized, possibly due to barriers in clinical adoption and accessibility [1].

Trend analysis showed that GLP-1 prescription rates fluctuated without a clear upward trajectory, with the highest rate observed in 2014 (0.22%) and the lowest in 2015 (0.06%). These low usage rates contrast with clinical guidelines that increasingly endorse GLP-1 agonists for their metabolic and cardiovascular benefits. Possible explanations include cost barriers, limited insurance coverage, the requirement for injectable administration, and provider unfamiliarity or reluctance to adopt newer pharmacologic agents during the study period.

Beyond GLP-1 use, other variables in our model offered insights into BMI variation. Older age was associated with slightly lower BMI, consistent with prior research suggesting that BMI may decline with age due to muscle loss or changes in body composition. Female patients and those with private or self-pay insurance had significantly lower BMI compared to their counterparts, which may reflect disparities in access to or utilization of obesity-related healthcare. Additionally, individuals with CVD exhibited higher BMI, emphasizing the interconnected burden of metabolic and cardiac conditions in this population. These findings align with existing literature on the interplay between insulin resistance, obesity, and chronic disease progression in type 2 diabetes, reinforcing the importance of addressing BMI in treatment strategies [3].

Obesity and CVD significantly worsen the challenges faced by individuals with T2DM. These comorbidities are interconnected, creating a complex network of healthcare challenges that reduce quality of life, increase healthcare costs, and heighten the risk of severe complications and premature death. GLP-1RAs offer therapeutic benefits by addressing the combined burdens of obesity and CVD in T2DM. Their effects extend across weight management, reduction in the risk of major adverse cardiovascular events, glycemic control, and anti-inflammatory actions on tissue [5].

GLP-1RAs have become a valuable pharmacologic tool for weight management in individuals with T2DM. Overweight and obesity are prevalent in this population, and even modest weight loss can improve glycemic control and reduce medication requirements. Castellana et al. highlighted the anorexigenic effects of GLP-1RAs, which promote satiety and suppress appetite, leading to reduced caloric intake. Their meta-analysis showed that GLP-1RAs, particularly when combined with SGLT2 inhibitors, significantly reduced body weight [12]. For example, liraglutide 3.0 mg resulted in a 6.4 kg weight loss in patients with T2DM and an 8.4 kg loss in obese individuals without diabetes. The analysis also found an additional 1.6 kg weight reduction when GLP-1RAs were combined with SGLT2 inhibitor therapy, potentially counteracting the increased appetite sometimes associated with SGLT2i use. Overall, GLP-1RAs provide dual benefits for glycemic control and weight reduction, supporting their growing role in obesity management among adults with T2DM.

GLP-1RAs provide cardiovascular benefits through multiple mechanisms, primarily by mitigating cardiovascular risk factors in T2DM. In addition to their well-established effects on glycemic control and weight management, GLP-1RAs have been shown to influence arterial hypertension [7]. Although the exact mechanism is not fully understood, one proposed pathway involves direct activation of GLP-1 receptors in the arterial and renal systems, resulting in vasodilation and a natriuretic effect via inhibition of the renin-angiotensin-aldosterone system [7]. GLP-1RAs may also act indirectly by stimulating nitric oxide production through cyclic GMP [21]. The antihypertensive effect of GLP-1RAs has been supported by clinical trials. In the LEAD trial, liraglutide reduced systolic blood pressure (SBP) by -2.6 to -6.6 mmHg [7,21,22]. Similarly, in the DURATION trial, weekly exenatide lowered SBP by -3 to -5 mmHg, particularly in patients with baseline SBP > 150 mmHg [23]. However, GLP-1RAs did not significantly affect blood pressure in normotensive patients [7].

GLP-1RAs have also demonstrated beneficial effects on lipid profiles, an important cardiovascular risk factor. In a meta-analysis of LEAD trials, liraglutide reduced LDL cholesterol by -7.73 mg/dL and total cholesterol by -5.03 mg/dL compared with standard therapy [24]. While the exact mechanism remains unclear, it has been suggested that improved glycemic control reduces insulin resistance and hepatic triglyceride synthesis, thereby improving lipid levels [7]. It is important to note that reductions in both SBP and cholesterol were secondary outcomes in these trials. Recent meta-analytic findings by Hu et al. [13] further highlight the multifaceted benefits of GLP-1RAs in T2DM, demonstrating significant decreases in systolic (-2.9 mmHg) and diastolic (-0.9 mmHg) blood pressure. Notably, weight loss emerged as the strongest predictor of blood pressure reduction, with each kilogram lost associated with a systolic decrease of β = 0.821 (P < 0.001). Glycemic control alone was not independently linked to blood pressure improvement, suggesting that GLP-1RAs exert their cardiometabolic benefits through both direct vascular effects and indirect weight-mediated pathways.

Despite these benefits, GLP-1RAs remain underprescribed. Our findings mirror this trend, showing more nonusers than users among obese patients with CVD and T2DM. Contributing factors likely include prescriber hesitancy, formulary restrictions, high out-of-pocket costs, limited insurance coverage, and clinical inertia. Notably, one study [22] reported that cardiologists often felt GLP-1RAs fell outside their prescribing scope, further limiting uptake. Additionally, disparities persist across racial and socioeconomic lines, as highlighted by Nathan et al. [16] in their analysis of DOAC prescriptions, a pattern that may extend to GLP-1RAs. Even when race is not a direct predictor, factors such as medication access and physician familiarity influence prescribing behavior. Some clinicians may also prefer combination therapy, as evidence suggests that GLP-1RAs combined with SGLT2 inhibitors improve both glycemic control and weight loss outcomes [12].

Looking beyond the study period, emerging therapies like tirzepatide, a dual GIP/GLP-1 receptor co-agonist, show promise in overcoming these barriers. The SURPASS trials demonstrated superior HbA1c reduction and weight loss with tirzepatide (up to 11.7 kg) compared with semaglutide and basal insulin [9]. However, barriers such as the long-term safety profile, cost, and limited availability may restrict wider adoption. Okerson et al. [23] also noted that severe nausea and vomiting can deter some patients, contributing to non-compliance and low initiation rates. Additionally, although rare, concerns about thyroid cancer risk may lead some physicians to avoid prescribing these agents initially [4].

Strengths and limitations

Our study has several strengths. It leverages the NAMCS, which employs a robust multistage sampling design and allows for nationally representative estimates. Additionally, the use of multiple years of data enabled the evaluation of temporal trends and adjustment for potential year effects. However, several important limitations should be considered when interpreting the findings. First, the data capture only prescription events and do not provide information on medication adherence, dosage, or duration of use, all of which could influence BMI outcomes. Second, only exenatide was identified in the dataset, which may not reflect the broader use or effectiveness of GLP-1RAs, especially given the limited uptake or availability of newer agents such as liraglutide and semaglutide during the study period. Third, the extremely low prevalence of GLP-1 use in the sample (0.13%) may have limited statistical power to detect associations or conduct subgroup analyses. While the observed association between GLP-1 use and BMI was statistically significant, the small number of exposed cases restricts the precision and generalizability of effect estimates. Finally, due to data availability, the analysis was limited to the years 2010 through 2015, preventing assessment of more recent prescribing trends or outcomes associated with newer GLP-1RAs. Future studies should consider longitudinal designs to clarify the temporal relationship between GLP-1 initiation and BMI changes, incorporate data on adherence and dosage, and utilize more recent datasets to evaluate the impact of currently available GLP-1 agents.

## Conclusions

This study found that, between 2010 and 2015, GLP-1 agonists were prescribed to only a small proportion of obese adults with type 2 diabetes in US outpatient settings. Notably, GLP-1 users had significantly higher BMI compared to nonusers, likely reflecting provider preference for prescribing these medications to patients with more severe obesity. These findings indicate underutilization of GLP-1RAs despite their well-documented benefits and underscore the need to improve access, enhance provider awareness, and promote guideline-based prescribing to optimize obesity and diabetes management.
